# FMRFamide-Related Peptides Signaling Is Involved in the Regulation of Muscle Contractions in Two Tenebrionid Beetles

**DOI:** 10.3389/fphys.2020.00456

**Published:** 2020-05-12

**Authors:** Paweł Marciniak, Wojciech Witek, Monika Szymczak, Joanna Pacholska-Bogalska, Szymon Chowański, Mariola Kuczer, Grzegorz Rosiński

**Affiliations:** ^1^Department of Animal Physiology and Development, Adam Mickiewicz University, Poznań, Poland; ^2^Faculty of Chemistry, University of Wrocław, Wrocław, Poland

**Keywords:** G-protein coupled receptor, visceral organs, FMRFamide-like peptides, neuropeptides, beetles (Coleoptera), heart

## Abstract

Peptidergic signaling regulates various physiological processes in insects. Neuropeptides are important messenger molecules that act as neurotransmitters, neuromodulators or hormones. Neuropeptides with myotropic properties in insects are known as FMRFamide-like peptides (FaLPs). Here, we describe the myotropic effects of the endogenous FaLPs in the regulation of contractile activity of the heart, ejaculatory duct, oviduct and the hindgut in two beetle species, *Tenebrio molitor* and *Zophobas atratus*. A putative receptor was identified *in silico* in both species. Using RT-PCR these putative FaLPs receptors were found in the various tissues of both beetles, including visceral organs. Analysis of the amino acid sequence of the receptor indicated that it is similar to other insect FaLPs receptors and belongs to G-protein coupled receptors. A synthetic FaLP (NSNFLRFa) found as the bioanalogue of both species demonstrated concentration-dependent and organ-specific myoactive properties. The peptide had species–specific cardioactivity, in that it stimulated *Z. atratus* heart contractions, while slightly inhibiting that of *T. molitor* and had mainly myostimulatory effect on the examined visceral organs of both beetle species, with the lowest activity in the ejaculatory duct of these beetles. The peptide was the most active in the hindgut of both species, but only at high concentration of 10^–5^ M. The results suggest that FaLPs are potent modulators of endogenous contractile activity of the visceral muscles in beetles and may indirectly affect various physiological processes.

## Introduction

FMRFamide is the four amino acid sequence (Phe-Met-Arg-Phe-NH_2_) first found as an molluscan cardioacceleratory agent (Price and Greenberg, 1977). FMRFamide itself is not present in insects, but the name has been used to describe peptides with a C-terminal RFamide motif, which were grouped together in a family of FMRFamide-like peptides (FaLPs) or FMRFamide-related peptides (FaRPs). The FaLPs “family” originally included N-terminally extended FMRFamides, myosuppressins (MS), neuropeptides F (NPF), and short neuropeptide F (sNPF). These peptides now are assigned to separate families, because they are encoded on different genes and have their own receptors. The insect FaLPs therefore comprises only the N-terminally extended FMRFamides (Coast and Schooley, 2011). A variable number of N-terminally extended FMRFamides are present in various insects along with extended IRF/Lamides (Coast and Schooley, 2011).

The physiological activity of insect FaLPs have been studied in different species and were shown to be mainly myoactive, however, with different effectivity. They regulate the heart and other visceral muscles contractility including male and female reproductive tracts (Merte and Nichols, 2002; Sedra and Lange, 2014; Suggs et al., 2016). Other known functions of FaLPs include involvement in regulation of circadian rhythm (Pyza and Meinertzhagen, 2003) and synaptic activity (Marques et al., 2003). Most of the research has been performed on different insect species, such as flies (Merte and Nichols, 2002), locusts (Robb and Evans, 1994), stick insects (Lange et al., 2009), or mosquitoes (Hillyer et al., 2014). However, scarce information is available regarding FaLPs in the largest insect order – the beetles. Thus far, only tetrapeptide FMRFa was shown to stimulate contractions of beetle reproductive tracts (Marciniak and Rosinski, 2010). Thus, the exact physiological role of FaLPs in beetles, as well as in other insects is still unclear.

**FIGURE 1:**
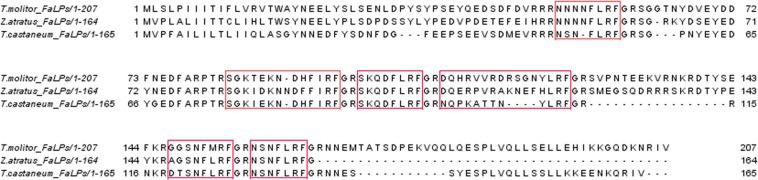
Sequence alignment of FaLPs precursors from *T. molitor* (*T. molitor*_FaLPs – this study), *Z. atratus* (*Z. atratus*_FaLPs – this study) and *T. castaneum* (*T. castaneum*_FaLPs, EFA02863), all from Tenebrionidae. Peptides present in the precursor are marked in red boxes.

Recently, due to the involvement of omics technologies, FaLPs precursors have been identified in several beetle species (Pandit et al., 2019). In all studied beetle species, the identified precursors contain several bioanalogues which the C-terminus contains either – FLRFa or – FIRFa (Veenstra, 2019). In tenebrionid beetles, FaLPs precursors’ sequences are available for three species: *Tenebrio molitor* and *Zophobas atratus* (this study) and the first beetle with complete neuropeptidome *Tribolium castaneum* (Li et al., 2008). In all of these species, the precursor contains six bioanalogues (Figure 1). To evaluate the organ-specific myotropic properties of FaLPs in beetles, here, we report the activity of the last bioanalogue from the precursor FMRF6 (NSNFLRFa) which was identical in all three species (Figure 1).

Most of the insect neuropeptides act *via* G protein coupled receptors (Audsley and Down, 2015). GPCRs have seven α-helical transmembrane domains and are, therefore, also called seven transmembrane (7TM) receptors and are one of the most common and important molecules in living organisms (Hauser et al., 2008). In beetles, a complete set of GPCRs for neuropeptides was first described in *T. castaneum* (Hauser et al., 2008). In this study, we predicted also the FaLPs receptor (FMRFR) in *T. molitor* and *Z. atratus* in order to evaluate whether the observed effects of peptide applications are direct due to ligand receptor interactions.

## Materials and Methods

### Insects

*Tenebrio molitor* adult males (4 weeks post-eclosion) were reared according to a previously described procedure (Rosinski et al., 1978). *Zophobas atratus* adults (4 weeks post-eclosion) were obtained from a colony maintained at the Department of Animal Physiology and Development, Adam Mickiewicz University, Poznań according to the Quennedy procedure (Quennedey et al., 1995).

### Peptides

Peptide FMRF6 (NSNFLRFa) was synthesized according to the Fmoc procedure, as described previously (Marciniak et al., 2008; Lubawy et al., 2018). Proctolin (RYLPT), used as a control peptide, was obtained from Sigma Aldrich (Germany). The peptides were dissolved in physiological saline appropriate for beetles (274 mM NaCl, 19 mM KCl, 9 mM CaCl_2_, 5 mM glucose, 5 mM, HEPES, pH 7.0) to yield a stock solution of 10^–3^ M, and it was stored at −20°C. Working dilutions were prepared from a stock solution in a physiological saline.

### Transcriptome Sequencing, Database Search, and Sequence Comparison

Transcriptomic data from *T. molitor* and *Z. atratus* were obtained after Illumina Hiseq sequencing of total RNA extracted from the brains (*n* = 10) and retrocerebral complexes (*n* = 10) of adult beetles and sequenced at the Beijing Genomics Institute (Shenzhen, China). After initial filtering of low quality reads and adaptor removal, clean reads were *de novo* assembled using Trinity. The transcriptomic data has been submitted to NCBI Sequence Read Archive database (SRR11184806 and SRR11358229, BioProject PRJNA608239 for *T. molitor* and SRR11178058 and SRR11178059, BioProject PRJNA608269 for *Z. atratus*) and were used for local tblastn withFaLPs precursor sequence from *T. castaneum* (EFA02863) and FaLPs receptor sequence from *T. castaneum* (NP_001280540.1) to find *T. molitor* and *Z. atratus* FaLPs precursor and receptor sequence.

### Receptor Transcript Distribution

Transcript profiles of Tenmo-FMRFR and Zopat-FMRFR were determined by reverse transcriptase PCR (RT-PCR) in various tissues of adults. RT-PCR was performed according to a modification of the method described by Marone et al. (2001). Suitable tissues/organs (nervous system – brain, retrocerebral complex CC/CA and ventral nerve cord, and heart, hindgut, ejaculatory duct and oviduct) after dissection were transferred to 150 μL of RNA lysis buffer (Zymo Research, United States) and homogenized for 3 min using a pellet homogenizer. The homogenized tissues/organs were immediately frozen in liquid nitrogen and then stored at –80°C. A Quick-RNA^®^ Mini Prep kit (Zymo Research, United States) was used for RNA isolation. The RNA concentration was determined with a Synergy H1 Hybrid Multi-Mode Microplate Reader (BioTek, United States). Reverse transcription of the same amount of isolated RNA to cDNA was accomplished using the RevertAid^TM^ Reverse Transcriptase kit (Thermo-Fisher Scientific, United States) according to the manufacturer’s protocol. PCR analyses were conducted using a T100^TM^ Thermal Cycler (Bio-Rad, United States). The primers were designed based on sequences of Tenmo-FMRFR and Zopat-FMRFR using Primer3 software (Untergasser et al., 2012). The primer pair for Tenmo-FMRFR was created to amplify fragments of 120 bp with the following sequences Fw 5′-AACATAATAGACACCTACTG-3′ and Rev 5′-CTTCTCACCGAATATCAC-3′, whereas the primers for Zopat-FMRFR amplify fragment of 143 bp and where Fw 5′-TACCTCCAGCTCTACCGCTT-3′ and Rev 5′-AGGCCGATGAGGAGGTAGTT-3′. The primers were synthetized by the Institute of Biochemistry and Biophysics of the Polish Academy of Science (Warsaw, Poland). PCR was performed in a 10 μL reaction volume containing 3.95 μL of DNase/RNase-free water, 1 μL of DreamTaq Green Buffer (Thermo-Fisher Scientific, United States), 1 μL of 2 mM dNTP, 1 μL of 10 μM forward primers, 1 μL of 10 μM reverse primers, 0.05 μL of DreamTaq DNA polymerase (Thermo-Fisher Scientific, United States) and 2 μL of cDNA. The obtained products were analyzed by electrophoresis using a 2% TAE agarose gel stained with ethidium bromide. The GeneRuler 100 bp DNA Ladder (Fermentas, United States) was run on each gel. Photos of the agarose gels were taken using ChemiDoc Touch (Bio-Rad, United States). PCR was with a minimum of five biological and three technical replicates. To confirm our results, the bands were sequenced with BigDye Terminator v3.1 on an ABI Prism 3130XL Analyzer (Applied Biosystems, Foster City, CA, United States) according to manufacturer’s protocols by the Molecular Biology Techniques Laboratory (Faculty of Biology, Adam Mickiewicz University, Poznań) and compared with transcriptomic data. “No template control” and “no RT control” reactions were included in the analysis to ensure that there was no foreign DNA or genomic DNA contamination.

### *In vitro* Visceral Organs Contraction Bioassays

The heart bioassay was performed by microdensitometric whereas the oviduct, ejaculatory duct and hindgut bioassay by a video microscopy techniques as described previously (Marciniak and Rosinski, 2010; Marciniak et al., 2011). In all bioassays, eight peptide concentrations were tested ranging from 10^–12^ to 10^–5^ M. Proctolin as a positive control in concentration 10^–7^ M was used (Supplementary Table S1). In brief, in the heart bioassay a semi-isolated heart preparations in superfusion chamber were mounted under the microdensitometer MD-100 (Carl Zeiss, Germany), whereas in video microscopy technique isolated visceral organs on Sylgard filled chamber were placed under the Olympus SZX12 stereomicroscope equipped with a SD30 camera. In all bioassays, an open perfusion system was used, with an injection port (for peptides) 70 mm above the superfusion chamber. The organ was subjected to a constant perfusion with fresh saline at the rate of about 140 μL/min. All tested samples were applied at the injection port with a Hamilton syringe. Many pulse applications of samples could be sequentially assayed in a single preparation. After the initial 15 min stabilization, the activity of the isolated organ was recorded for 2 min. Next the peptide was applied and the heart activity was recorded for a further 2 min. In the heart bioassay, the apparatus equipped with photocell counts optically every contraction of the heart, and being connected with microdensitometer register system, computer generated the cardiogram. In the video microscopy technique, first the video recordings of superfused organs were performed and then analyzed with the edge tracking software (AnTracker) to create a trace of the movement of the side edge of the organ.

### Statistics

All statistical comparisons non-parametric *t*-tests (Mann–Whitney test) were performed with usage of Graph Pad Prism 6 software (AMU license). Results were considered statistically significant with *p* < 0.05. Prior to the analysis Shapiro-Wilk normality test was done for all of the groups.

## Results

### Analysis of *T. molitor* and *Z. atratus* FaLPs Precursor and FMRFR Sequences

A blast search in the transcriptomes assemblies of *T. molitor* and *Z. atratus* yielded the FaLPs precursors of *T. molitor* and *Z. atratus*. Precursors were similar in structure to the homologous precursor from *T. castaneum* and encode six peptides of which two are identical in all of the beetles (Figure 1).

**FIGURE 2:**
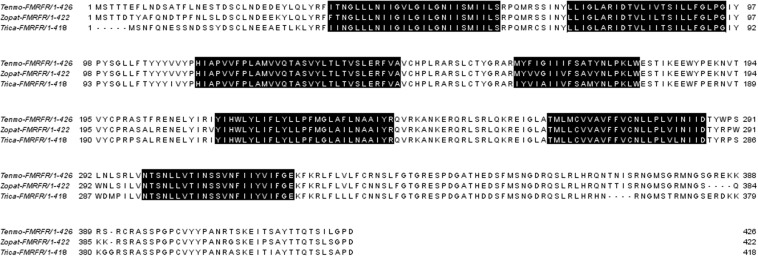
Alignment of the FMRFRs sequences from *T. molitor* (Tenmo-FMRFR), *Z. atratus* (Zopat-FMRFR), and *T. castaneum* (Trica-FMRFR), all from the Tenebrionidae family. Predicted seven transmembrane domains are highlighted in black.

The BLAST search with local databases of the transcriptomic assemblies of *T. molitor* and *Z. atratus* brains and retrocerebral complexes yielded one open reading frame of a putative FaLPs receptor in each assembly. The receptor in *T. molitor* is 1,278 bp and in *Z. atratus* is 1,266 bp (Figure 2). Both display the seven transmembrane domains typical for GPCRs (Bass et al., 2014) with an N-terminal ligand binding region and a C-terminal intracellular region. The comparison of the putative sequences showed that there is a very high degree of similarity (90%) between Tenmo-FMRFR and Zopat-FMRF. Less but also very high similarity was observed between Tenmo-FMRFR and Trica-FMRFR –84% and between Zopat-FMRFR and Trica-FMRFR –86%.

### Distribution of FMRFR Transcripts in Different Tissues of *T. molitor* and *Z. atratus*

**FIGURE 3:**
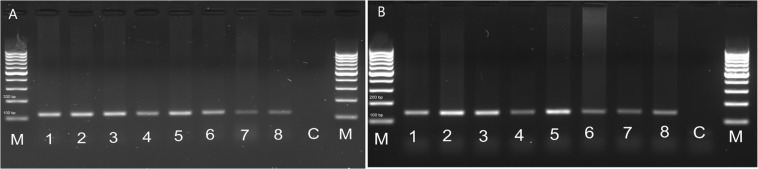
Distribution of FMRFR transcript in different tissues of *T. molitor* **(A)** and *Z. atratus* **(B)**. 1, whole body; 2, brain; 3, ventral nerve cord; 4, retrocerebral complex; 5, heart; 6, hindgut; 7, ejaculatory duct; 8, oviduct; C, control H_2_0; M, marker.

In order to check whether the peptide is able to influence the visceral organs directly, we examined the FMRFR spatial distribution by RT-PCR. As a positive control, we used nervous tissues, as FMRFR was previously shown to be present in this tissue in other insects, such as *D. melanogaster* (Meeusen et al., 2002). Analysis of FMRFR transcripts distribution in both species revealed that it is present in all tissues used for RNA isolation. It proves that FMRFR is present in the nervous system as well as various visceral organs of tenebrionid beetles (Figure 3). Despite the fact that quantitative analysis was not performed, the band intensity indicates that the level of the transcript may vary between tissues tested and, as expected, is the highest in the nervous system of both beetles and much lower in the digestive tract and the reproductive tract (Figure 3).

### Effect of FMRF6 on the *T. molitor* Visceral Muscles Contractility

**FIGURE 4:**
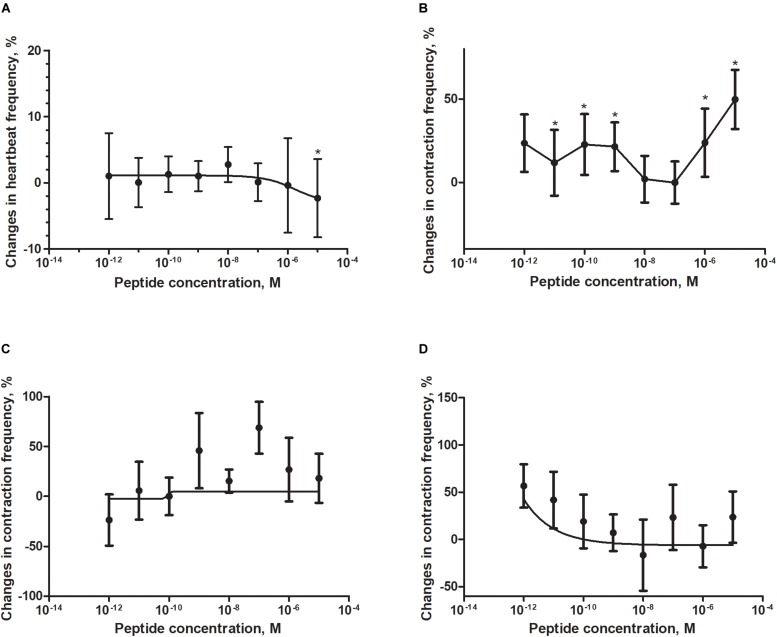
Changes compared to control in the contractions frequency of heart **(A)**, hindgut **(B)**, ejaculatory duct **(C)**, and oviduct **(D)** of adult *T. molitor* beetle after application of FMRFa. Mean ± SEM are given from at least eight determinations. Statistically significant differences (*p* ≤ 0.05) in the contractions frequency from control (saline application) are indicated by asterisks (*t*-Student test) *n* = 10–15.

Application of a synthetic tenebrionid FMRF6 peptide during superfusion with physiological saline caused differentiated effects in frequency of contractions of the heart, hindgut, ejaculatory duct and oviduct of *T. molitor* beetle (Figure 4). The observed effects were organ-specific.

The *T. molitor* adult heart rhythm *in vitro* remained regular during superfusion with saline and showed on average 124 beats/min. Application of FMRF6 caused immediate, dose-dependent and reversible slight decrease of the heart contractile activity. The significant cardioinhibition was caused only by the highest tested concentration – 10^–5^ M (Figure 4A). All other concentrations from 10^–12^ to 10^–6^ M caused no effects.

**FIGURE 5:**
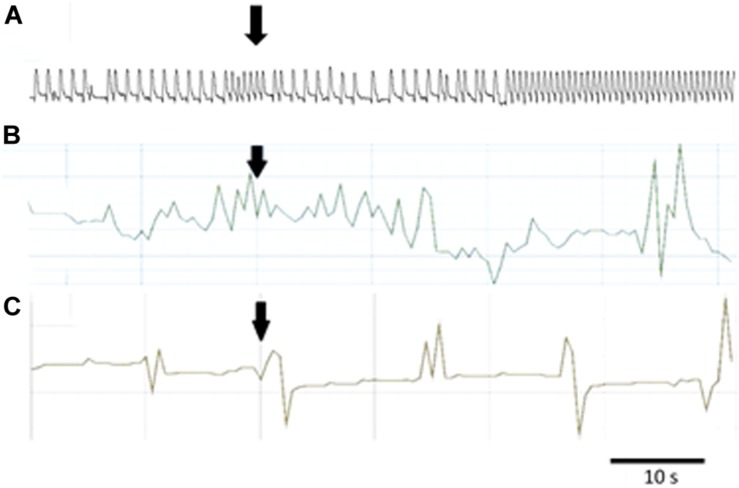
Typical responses in the contraction frequency of *Z. atratus* heart after application of FMRF6 at the concentration 10^–5^ M **(A)**; *T. molitor* hindgut at the concentration 10^–6^ M **(B)**; *Z. atratus* oviduct at the concentration 10^–6^ M **(C)**. Peptide application is indicated by an arrow.

The hindgut contractions of adult *T. molitor* remained irregular during superfusion with saline (5 contractions/min on average). Contrary to the heart, application of the FMRF6 peptide to the hindgut caused an increase in contraction frequency (Figures 4B, 5B) in almost all of tested concentrations apart from 10^–8^ to 10^–7^ M. The highest myotropic effect (51% stimulation) was observed after application of the highest concentration 10^–5^ M. Remarkably in lower concentrations (10^–12^–10^–11^ M) less potent myostimulation also was observed with an effect half that observed after 10^–5^ M application.

The *T. molitor* ejaculatory duct contractions were very irregular during superfusion with physiological saline with the level of 2 contraction/min on average (Figure 4C). Application of FMRF6 peptide caused no statistically significant effects in the contractions frequency, however in single repetitions stimulation of 70% was sometimes observed.

Contrary to the ejaculatory duct, the oviduct of *T. molitor* females contracted more regular with an average of 7 contractions/min (Figure 4D). FMRF6 caused dose-dependent miostimulatory effects on the oviduct. Similarly to the hindgut bioassay, the highest increase in contraction frequency (50%) was observed in lower concentrations (10^–12^–10^–11^ M). In higher concentration ranges, the stimulatory effect was not so evident.

### Effect of FMRF6 on the *Z. atratus* Visceral Muscles Contractility

**FIGURE 6:**
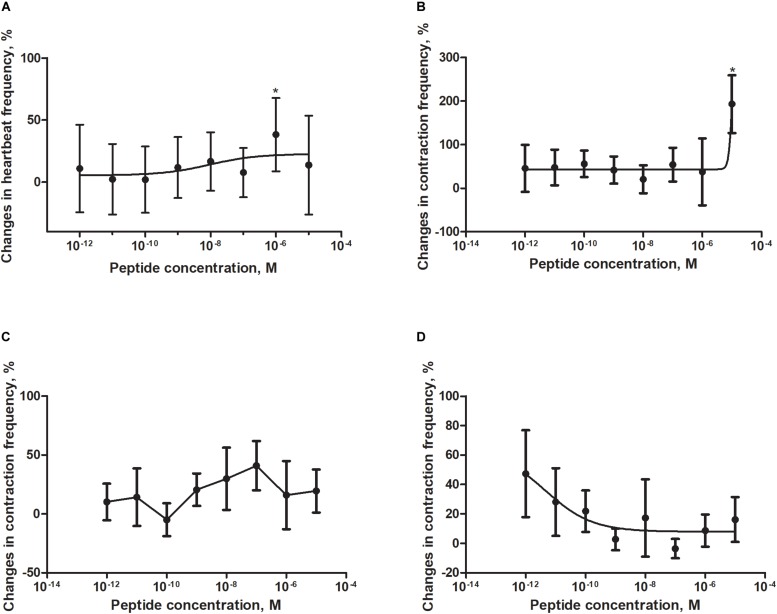
Changes compared to control in the contractions frequency of heart **(A)**, hindgut **(B)**, ejaculatory duct **(C)**, and oviduct **(D)** of adult *Z. atratus* after application of FMRF6. Mean ± SEM are given from at least eight determinations. Statistically significant differences (*p* ≤ 0.05) in the contractions frequency from control (saline application) are indicated by asterisks (*t*-Student test).

In *in vitro* bioassays with isolated *Z. atratus* visceral organs, peptide FMRF6 caused differentiated myotropic effects. Similar to other insect evaluated, the effect of FMRF6 in *Z. atratus* was organ-specific (Figure 6).

Semi-isolated heart of adult *Z. atraus* during superfusion with physiological saline showed regular rhythm with an average 35 beats/min, which was much slower than that of *T. molitor* (Figure 5A). In contrast to *T. molitor*, addition of the peptide resulted in a concentration-dependent positive chronotropic effects (increase in heartbeat frequency). The maximal effect was observed after application of one of the highest concentrations 10^–6^ M, as was shown for the previously described species, where also only the highest concentrations were cardioactive (Figure 6A).

The highest tested concentration (10^–5^ M) also was myostimulatory when applied on the isolated hindgut of *Z. atratus*. Average contraction frequency of the hindgut during superfusion was around 10 and increased to 193% after its application. Remarkably all other concentrations tested (10^–12^–10^–6^ M) showed also an increase in contraction frequency of on average 37% (Figure 6B).

The irregular control ejaculatory duct contraction during superfusion with saline was on average 3 contractions/min. Similar to *T. molitor*, application of FMRF6 resulted in an increase in contraction frequency but the effect was much stronger than in *T. molitor*. The highest increase in contraction frequency (48%) was measured after application of 10^–7^ M. All tested concentrations between 10^–9^ and 10^–6^ M were myostimulatory with different efficiency (Figure 6C). Only the concentration of 10^–10^ M was inactive when applied on the oviduct (Figure 6C).

The last tested visceral organ – the oviduct from *Z. atratus*, had a control contraction rhythm at the level of 13 contractions/min on average. Similar to *T. molitor*, application of FMRF6 caused an increase in the contraction frequency but only in the lower tested concentration ranges (Figure 6D). The strongest effect was obtained after application of the lowest 10^–12^ M concentration and the increase in contractions frequency was about 47%. The highest concentrations from 10^–9^ to 10^–5^ M caused no effect (Figure 5C).

## Discussion

The present study expands the knowledge about the physiological role of FaLPs in the largest group of insects – Coleoptera. Here, we have examined the role of a N-terminally extended bioanalogue of FMRFa in the regulation of visceral organs contractions in *T. molitor* and *Z. atratus* beetle. We used a synthetic peptide FMRF6 (NSNFLRFa), which was identical in the FaLPs precursors of both tested beetles. We showed in *in vitro* bioassays, that the peptide was mainly myostimulatory and, with the exception of the *T. molitor* heart, it increased the frequency of contractions of beetles muscles organs with different efficiency. The effects appear to be organ-specific. An oviduct of both beetle species turned out to be the most sensitive to the peptide. The peptide acts *via* the putative GPCR characterized in both species.

FaLPs signaling has been studied in variety of insect species, including mosquitoes (Hillyer et al., 2014), stick insects (Lange et al., 2009), kissing bugs (Sedra and Lange, 2014), or flies (Merte and Nichols, 2002). However, the receptor for FaLPs was first discovered (Cazzamali and Grimmelikhuijzen, 2002) and very well characterized only in *D. melanogaster* (Maynard et al., 2013; Milakovic et al., 2014). As for most of the receptors activated by neuropeptides, FMRFRs belong to G-protein coupled receptors with seven transmembrane helixes, N-terminal extracellular segment and the intracellular C-terminus responsible for interactions with G proteins (Hauser et al., 2008). Moreover, a detailed analysis of insect FMRFR indicated that all belong to the subfamily A – rhodopsin like GPCRs thus far identified in flies, mosquitoes, moths and kissing bugs (Rasmussen et al., 2015). In beetles, the first FMRFR was identified in the model species for this order – *T. castaneum* (Hauser et al., 2008). Also, the receptor predicted in this study resembles this type of receptors containing some of typical rhodopsin-like amino acid patterns in its seven transmembrane domains: GN in helix 1, NLX3-DX8P in helix 2, SX3LX2IX2DRY in helix 3, WX8P in helix 4, FX2PX7Y in helix 5, FX3WXP in helix 6 and NPX2YX6F in helix 7 (Figure 2; Bass et al., 2014). Bioinformatical analysis demonstrated that the sequence similarity within tenebrionid beetles as expected is high.

The spatial tissue distribution of FMRFR was first studied in *D. melanogaster* (Meeusen et al., 2002). These authors showed that Drome-FMRFR is present in the nervous system (brain), guts and ovaries of larva and adult *D. melanogaster*, as well as trachea and fat body (Meeusen et al., 2002), suggesting that FaLPs might be involved in regulation of various physiological processes and in agreement with our study. We showed that the FaLPs receptor predicted here is present in the nervous system (brain, ventral nerve cord and retrocerebral complex CC/CA) as well as in all tested visceral organs suggesting the role of FaLPs in the regulation of functions of these organs. The data also is partially in agreement with studies performed on mosquitoes (Hillyer et al., 2014). The highest expression of FMRFR in *Anopheles gambiae* occurred in the head and thorax of this insects (Hillyer et al., 2014). It is surprising that in *A. gambiae* the expression level of FMRFR was so low in the abdomen. As in numerous insects, FaLPs immunoreactivity was observed in abdominal ganglia, midguts, and other visceral organs as well as in abdominal neural processes in *D. melanogaster*, *R. prolixus*, *Schistocerca gregaria*, *Phormia regina* (blow fly) and other insects (Myers and Evans, 1985; Nichols et al., 1995; Sedra and Lange, 2014), and therefore it was expected that the FaLPs receptors will be highly expressed in the abdomen. Probably this is the case in *D. melanogaster* and in the beetles studied here but not in the mosquito. This differences might be connected with role of FaLPs in visceral organs physiology. These beetles and mosquitos differ significantly in how they ingest and process food.

FaLPs are neuropeptides which are known to be mainly myoactive (Merte and Nichols, 2002). The best established activity of these peptides is their involvement in the regulation of the heart rhythm (Chowanski et al., 2016). The cardioactive properties of different FaLPs were shown in variety of insect species including *S. gregaria, B. extradentatum*, *D. melanogaster* and *A. gambiae* (Cuthbert and Evans, 1989; Nichols, 2006; Lange et al., 2009; Hillyer et al., 2014). These studies have not been performed on beetles before now. Most insect species exhibit dose-dependant cardioacceleratory properties and our study is partially in agreement. FMRF6 was cardio acceleratory on the heart of *Z. atratus*. Surprisingly the same peptide was cardio inhibitory on the *T. molitor* heart in the high concentration ranges. In the very high concentrations (10^–3^–10^–2^ M), one of the N-terminally extended FaLPs was also cardioinhibitory in mosquitoes (Hillyer et al., 2014). However, in *A. gambiae* a FaLP peptide exerted bimodal effect. It was cardio acceleratory in low concentrations whereas in our study it exerted no cardiotropic effects.

Apart from the heart, FaLPs were shown to be myoactive on different visceral organs such as the gut and reproductive tract (ejaculatory duct and oviduct) of tenebrionids. As in the heart, the peptide was shown to be mostly myostimulatory. The results obtained in this study are in agreement with results obtained in other species, especially the extensively studied female reproductive tract. It was shown for example that various parts of the *R. prolixus* reproductive tract are stimulated by N-terminally extended FaLPs and the effect is similar to the FMRFa peptide (Sedra and Lange, 2014, 2016). In beetles, no previous studies were available for FaLPs in terms of the regulation of contractility of visceral organs. Previous studies examined only the tetrapeptide FMRFa on the motility of *T. molitor* and *Z. atratus* ejaculatory duct and oviduct (Marciniak and Rosinski, 2010). We showed that contractions of ejaculatory duct was inhibited whereas oviduct contractions were mainly stimulated after application of FMRFa in these tenebrionids. In both cases, the effects were bimodal and dose-dependent (Marciniak and Rosinski, 2010). The different responses of the ejaculatory duct and oviduct to F**M**RFa and FMRF6 (NSNF**L**RFa) is probably due to difference in amino acid – M changed to L, which affects receptor binding.

In summary, we demonstrated the myostimulatory activity of N-terminally extended FaLP in two tenebrionid beetles. The tested peptide NSNFLRFa is present in both insects in the sequence of the FaLPs precursor and presumably act via G-protein coupled receptors, which were also predicted and identified in both species. We showed that in both beetles this peptide is myostimulatory to the gut, ejaculatory duct and oviduct and the effect is dose-dependent in almost all cases. The data suggest that FaLPs regulate functioning of this organs thus are involved in different physiological processes. The role of FaLPs in cardiac physiology is more complex. The effect of peptide is dose-dependent and species specific.

## Data Availability Statement

The transcriptomic data has been submitted to NCBI Sequence Read Archive database (SRR11184806 and SRR11358229, BioProject PRJNA608239 for *T. molitor* and SRR11178058 and SRR11178059, BioProject PRJNA608269 for *Z. atratus*).

## Author Contributions

PM and GR contributed conception of the study. PM designed the study, analyzed the data, and wrote the manuscript. PM, WW, and MS performed the physiological experiments. JP-B and SC performed the molecular experiments. MK performed the peptide synthesis. All authors contributed to manuscript, read and approved the submitted version.

## Conflict of Interest

The authors declare that the research was conducted in the absence of any commercial or financial relationships that could be construed as a potential conflict of interest.
