# Supplementary material for: FMRFamide-Related Peptides Signaling Is Involved in the Regulation of Muscle Contractions in Two Tenebrionid Beetles
Source: Front Physiol. 2020 May 12;11:456. doi: 10.3389/fphys.2020.00456 (PMC7235380; doi:10.3389/fphys.2020.00456)
Supplement: Supplementary file 1 [file Table_1.DOCX]

Supplementary material 1

Increase compared to control in the contractions frequency of visceral organs of tenebrionid beetles after application of proctolin in concentration 10^-7^ M, which was used as a control in myotropic experiments.

|  | ***Tenebrio molitor*** | ***Zophobas atratus*** |
| --- | --- | --- |
| **Organ** | **Changes in contraction frequency, %** | |
| heart | 36±5 | 41±5 |
| hindgut | 162±643 | 137±117 |
| ejaculatory duct | 153±149 | 75±99 |
| oviduct | 109±158 | 140±170 |
